# Relationship between plasma lipids and mild cognitive impairment in the elderly Chinese: a case-control study

**DOI:** 10.1186/s12944-016-0320-6

**Published:** 2016-09-05

**Authors:** Qian He, Qing Li, Jiangang Zhao, Tianfeng Wu, Lu Ji, Guowei Huang, Fei Ma

**Affiliations:** 1 Department of Laboratory, General Hospital of Tianjin Medical University, Tianjin, 300052 China; 2 Department of Epidemiology and Biostatistics, School of Public Health, Tianjin Medical University, No 22 Qixiangtai Road, Heping District, Tianjin, 300070 China; 3 Department of Nutrition and Food Science, School of Public Health, Tianjin Medical University, Tianjin, 300070 China

**Keywords:** Mild cognitive impairment, Lipid, Lipoprotein, Elderly, Case-control study

## Abstract

**Background:**

High lipid levels may constitute a more important risk factor for cognitive health in previous studies. However, the association of plasma lipids with mild cognitive impairment (MCI) among elderly people had not been studied exactly. This study aims to explore the relationship between plasma lipids/lipoproteins and the risk of MCI in elderly Chinese individuals.

**Methods:**

CSI-MCI study was a preliminary case-control study of the association of plasma lipids/lipoproteins with MCI in 112 MCI cases and 115 cognitively normal controls. Plasma total cholesterol (TC), high-density lipoprotein cholesterol (HDL-C), low-density lipoprotein cholesterol (LDL-C), and triglycerides (TG) levels were measured in fasting blood samples. Multivariable logistic regression was used to evaluate the potential association between MCI and these factors. Statistical models were adjusted for multiple demographic and biological covariates.

**Results:**

The subjects with MCI were significantly older, higher percentage of females and less educated than controls (*P* <0.05). As expected, subjects with MCI had lower MMSE score compared with controls (*P* <0.05). Multivariate logistic regression analysis showed that higher plasma TC level was associated with the risk of MCI in models adjusting for age, sex and education. However, This association was attenuated after adjusting for BMI, Type 2 diabetes mellitus, heart disease and hypertension. Plasma TG level was negatively associated with the risk of MCI. The adjusted odds ratio (OR) of MCI was significantly reduced for the highest quartile of plasma TG level (OR: 0.76, 95 % CI: 0.48–0.97), but not for the second or third quartile, compared with the lowest quartile (adjusted models). Plasma HDL level was significantly negatively associated with the risk of MCI. There was no association between plasma LDL level and the risk of MCI, adjustment for demographics, vascular disorders did not change this relation.

**Conclusions:**

Plasma TC was significantly higher in MCI subjects compared to cognitively normal controls, Elevated plasma HDL and triglyceride were associated with the occurrence of MCI. These findings need to be confirmed in further longitudinal studies.

## Background

Mild cognitive impairment (MCI) is regarded as a transition stage between the cognitive changes of normal aging and the more serious problems caused by Alzheimer’s disease (AD) [1]. Persons with MCI convert to AD at an annual rate of 10–12 % in contrast to 1–2 % in the elderly population without MCI [2]. Early diagnosis and intervention of MCI could postpone or prevent the onset of subsequent dementia. It is critical to identify potentially protective factors for the development of MCI and progression to dementia. There is an important clinical need for diagnostic biomarkers to identify geriatric individuals prone to dementia. In recent decades, efforts have been made to discover biological markers for dementia and MCI.

Blood-based (non-genetic) biomarkers are important because they are easily acquired, relatively inexpensive compared to brain imaging biomarkers, less invasive than cerebrospinal fluid (CSF) acquisition, and more amenable to large-scale screening. Lipids and lipoproteins may directly affect neurodegeneration [3–6]. There are numerous studies investigating the relation between plasma lipid/lipoprotein levels and dementia. Several studies show that elderly people with AD or with dementia or cognitive deficits have higher plasma total cholesterol (TC) or higher low-density lipoprotein cholesterol (LDL-C) than sex- and age-matched nondemented peers [7–9]. Others fail to find such differences [10–12], and still others find negative correlations of serum lipid values with AD (or with all dementias) [13, 14]. The discrepancy in the results of a significant number of recent studies indicates that further investigation of lipid parameters is required, particularly in view of their increased biological importance and the ease of evaluation in everyday practice.

In this context, using a retrospective study design, we aimed to explore whether plasma lipid levels are associated with the risk of MCI in an elderly, community-dwelling population. Furthermore, we explored whether observed associations between individual components of the adverse lipid profiles and risk of MCI were dependent on or independent of demographics and vascular disorders.

## Methods

### Study design and participants

**Fig. 1:**
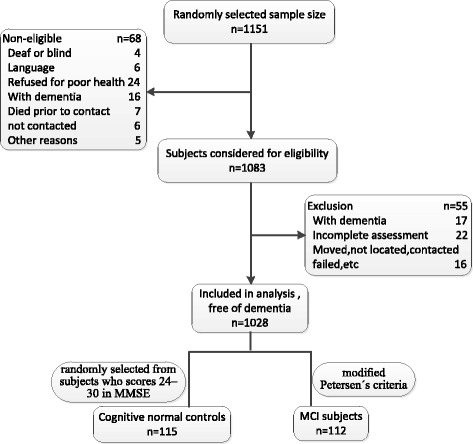
Formation of the study population

The Community Screening Interview for MCI (CSI-MCI) study was a population-based case-control study of comparing MCI subjects with cognitively normal controls. 1151 subjects 65 years or older were randomly selected using the medical records-linkage system of Binhai New Area in Tianjin, China. Each participant underwent an interview of general health and function, medical history, and a neuropsychological battery. Subjects with a preexisting diagnosis of dementia were identified by screening their medical record, and the clinical information was reviewed in detail by a neurologist (Dr Jiangang Zhao). Subjects confirmed to have dementia were not invited to participate in the study. A total of 1028 subjects without dementia were included in the active evaluation. The evaluations was conducted by trained graduate students and mental health clinicians, and conducted from April, 2014 to July, 2014. Among 1028 eligible participants, 112 had MCI. Detailed screening process can be seen in Fig. 1.

### Definition of cases and controls

MCI was identified according with the modified Petersen’s criteria [15]:

1. Subjective memory complaint, with at least 2-week duration, was reported by the participant and corroborated by an informant (family or physician);
2. Objective memory impairment for age and education has been defined by performing at least 1.5 SD below age and education-matched controls on memory subtask of Mini-Mental Status Examination (MMSE) [16];
3. Normal general cognitive function impairment was defined as a test performance more than 1.5 SD below age- and education-specific norms;
4. Essentially preserved activities of daily living, as measured by Activities of Daily Living scale (ADL), i.e. a score <26 [17];
5. Absence of dementia (Diagnostic and Statistical Manual of Mental Disorders-IV), AD (the National Institute of Neurological and Communicative Disorders and Stroke and the Alzheimer’s Disease and Related Disorders Association), or psychiatric disorders, cerebral damages, or physical diseases that may account for cognitive impairment, and any active neuropsychiatric condition producing disability.

Cognitive normal controls were screened by using MMSE tool. MMSE scores ranged from 0 to 30, with higher scores indicating better cognition. Cognitive function was classified into four groups based on the standard classification system [18]:no cognitive impairment (scores 24–30); mild cognitive impairment (scores 18–23); moderate cognitive impairment(scores 10–17) and severe cognitive impairment (scores 0–9). Cognitive normal controls were randomly selected from subjects who scores 24–30 in MMSE. Control subjects had no cognitive complaints, no evidence of impairment in the ADL due to cognitive disturbances, In both groups, persons having had a general anesthesia in the last 6 months, history of neurological diseases or events (i.e. Parkinson’s disease, epilepsy, brain anoxia), psychiatric disorder (schizophrenia, major depression, alcoholism) or traumatic brain injury were excluded from this study. Presence of other chronic diseases was not a reason for exclusion.

### Questionnaire survey and health examination

Structured interviews were carried out with all participants face-to-face. A variety of potential risk factors for cognitive function were included in the questionnaire. Covariates included sociodemographic information [age, gender, years of education and body mass index (BMI)], lifestyle (smoking and drinking). BMI was calculated as weight in kilograms divided by the square of height in meters. Type 2 diabetes mellitus(T2DM) were defined as a history of either disorder provided by patients at any time during life and confirmed by clinical evaluation. If affirmed, they were asked whether they were under treatment and the specific type of medication. Hypertension was defined as SBP > 140 mmHg, DBP > 90 mmHg, or the use of antihypertensive medication. All pharmacological treatments received during the month preceding the interview were recorded. Medical prescriptions and, when feasible, the medications were checked by the interviewer. The presence of stroke was ascertained from an interview with participants and their informants. When available, previous medical records were reviewed. Heart disease was defined as a history of myocardial infarction, congestive heart failure, digitalis use, or angina pectoris at any time during life. Whenever possible, these comorbidities were confirmed with information from the medical records-linkage system.

### Determination of plasma lipids

Blood samples were obtained from each participant after 12-h overnight fasting by standard venipuncture. kept at 4 °C for 1 h, and centrifuged at 3000 rpm for 10 min at 4 °C to obtain plasma and stored at −80 °C until analysis.

Plasma TC (normal range: 80–220 mg/dl) and Triglyceride(TG) (30–200 mg/dl) concentrations were measured by an Automatic Biochemistry Analyzer (Hitachi 7180, Japan) using commercially available diagnostic kits (Roche Diagnostic, Mannheim, Germany). High-density lipoprotein cholesterol (HDL-C) (normal range: 35–80 mg/dl) concentrations was measured with a Hitachi 911 autoanalyzer. LDL-C (30–160 mg/dl) was estimated with the Friedenwald equation [19]: LDL-C = TC − (HDL-C) − (TG/5). Concentrations of plasma lipids and lipoproteins were grouped into four equal strata representing decreasing concentrations of lipids and lipoproteins. All laboratory analyses were conducted by the central lab in school of public health, Tianjin Medical University in Tianjin, China.

### Statistical analysis

Lipid levels and other potentially relevant factors were compared among individuals with MCI and cognitively normal controls. Mann–Whitney or *t*-test for continuous variables and *χ*^*2*^ test for categorical variables (or trend tests if applicable) were used to test for significant differences between the groups. Because the distribution of HDL-C and TG levels was skewed, we performed logarithmic transformation of these data and repeated the statistical tests. Multivariate logistic regression was used to estimate the odds ratio (OR) of MCI associated with plasma lipid levels. ORs and 95 % confidence intervals (95 % CIs) were calculated. After adjusting for sex, age, and education, we performed a second model adjusting for BMI, T2DM, hypertension, and heart disease. *P* < 0.05 was considered statistically significant, and all *P*-values were two-sided. All analyses were performed using SPSS PASW Statistics for Windows, version 18.0 (SPSS Inc., Released 2009, Chicago, IL, USA).

## Results

### The principal characteristics of the two groups

This study included a total of 227 participants, including 112 persons with MCI and 115 cognitively normal controls. Lipid levels, demographics, and vascular risk factors were compared among individuals with MCI and controls in univariate analyses (Table 1). The subjects with MCI were significantly older, higher percentage of females and less educated than controls. As expected, subjects with MCI had lower MMSE score compared with controls. No significant differences were found between MCI subjects and controls for current smoking. TC concentration was higher in MCI compared with controls (*P* <0.05), while HDL-C and TG was lower in MCI compared with controls (*P* < 0.05). No statistical significance was found for LDL-C data between MCI and control groups. A history of T2DM, heart disease, and hypertension was more frequent in the MCI groups compared with the control group (*P* <0.05).

**Table 1:** Principal characteristics and lipid profile of MCI and cognitively normal controls

Characteristics	MCI group (*n* = 112)	Control group (*n* = 115)	*P* value
Age, years (Mean ± SD)^b^	76.32 ± 5.73^*^	74.51 ± 6.02	0.023
Female gender (%)^a^	79 (70.54)^*^	55 (47.83)	0.002
Education, years (Mean ± SD)^b^	8.62 ± 4.63^*^	9.12 ± 4.51	0.017
MMSE scores (Mean ± SD)^b^	17.25 ± 4.28^*^	25.22 ± 3.25	0.001
BMI (kg/m^2^) (Mean ± SD)^b^	25.51 ± 4.62^*^	27.41 ± 5.41	0.004
Current smoking (%)^a^	11 (9.82)	13 (11.30)	0.783
Lipid level (mg/dl)^b^			
TC (Mean ± SD)	196.61 ± 17.22^*^	190.21 ± 20.92	0.012
TG (Mean ± SD)	147.53 ± 27.52^*^	156.52 ± 28.51	0.018
HDL-C (Mean ± SD)	46.72 ± 5.51^*^	48.61 ± 5.82	0.014
LDL-C (Mean ± SD)	118.62 ± 34.31	120.22 ± 36.41	0.742
Vascular disorders (%)^a^			
Stroke	19 (18.96)	15 (7.04)	0.321
T2DM	32 (28.57)^*^	17 (14.78)	0.013
Hypertension	73 (65.18)^*^	48 (41.74)	0.001
Heart disease	42 (37.50)^*^	27 (23.48)	0.031

Figures in parentheses indicate percentages; Values for continuous variables are mean ± standard deviation

*Abbreviations*: *BMI* Body Mass Index, *HDL-C* High-density lipoprotein cholesterol, *LDL-C* Low-density lipoprotein cholesterol, *T2DM* diabetes mellitus type II, *TC* Total Cholesterol, *TG* Triglyceride

*significant at *P* < 0.05 vs control group

^a^Dichotomous variables (n, %) were tested using *χ* ^*2*^ test

^b^Continuous variables (mean, SD) were tested for differences between the two groups using Mann–Whitney or *t*-test

### Multivariate logistic regression analysis

In Table 2 are reported the results of multivariate logistic regression analysis assessing the effect of plasma lipids and other variables on the likelihood of having MCI. Higher plasma TC level was associated with the risk of MCI in models adjusting for age, sex and education. However, this association was attenuated after adjusting for BMI, T2DM, heart disease and hypertension. Plasma TG level was negatively associated with the risk of MCI. The adjusted OR of MCI was significantly reduced for the highest quartile of plasma TG concentration (OR: 0.76, 95 % CI: 0.48–0.97), but not for the second or third quartile, compared with the lowest quartile (adjusted models). Plasma HDL level was significantly negatively associated with the risk of MCI. There was no association between plasma LDL level and the risk of MCI, adjustment for demographics, vascular disorders did not change this relation.

**Table 2:** Multivariate logistic regression analysis exploring association between quartiles of plasma lipid levels and the risk of MCI after adjustment for demographics, vascular disorders

Lipid, Quartile (Range, mg/dl)	No. (%) of subjects		Basic Model^a^	Final Model^b^
	MCI group (*n* = 112)	Control group (*n* = 115)		
TC				
1 (≤172.00)	31 (27.7)	28 (24.3)	1.00	1.00
2 (172.01–197.00)	33 (29.5)	29 (25.2)	1.52 (1.08–2.32)	1.39 (1.07–2.11)
3 (197.01–225.00)	25 (22.3)	27 (23.5)	1.99 (1.47–2.81)	1.79 (1.33–2.54)
4 (≥225.01)	23 (20.5)	31 (27.0)	2.72 (1.82–3.81)	2.65 (2.13–3.39)
Trend test			*P* = 0.042	*P* = 0.031
HDL-C				
1 (≤37.00)	34 (30.3)	27 (23.5)	1.00	1.00
2 (37.01–45.00)	22 (19.6)	31 (27.0)	0.62 (0.45–0.91)	0.66 (0.41–1.08)
3 (45.01–55.00)	24 (21.4)	28 (24.3)	0.56 (0.45–0.97)	0.61 (0.38–0.98)
4 (≥55.01)	32 (28.6)	29 (25.2)	0.48 (0.23–0.73)	0.56 (0.37–0.83)
Trend test			*P* = 0.040	*P* = 0.033
TG				
1 (≤98.25)	33 (29.9)	30 (26.1)	1.00	1.00
2 (98.26–142.50)	30 (26.6)	28 (24.3)	0.85 (0.57–1.27)	0.95 (0.58–1.56)
3 (142.51–197.50)	25 (22.3)	32 (27.8)	0.82 (0.54–1.26)	0.93 (0.58–1.50)
4 (≥197.51)	27 (24.1)	25 (21.7)	0.65 (0.43–0.91)	0.76 (0.48–0.97)
Trend test			*P* = 0.047	*P* = 0.039
LDL-C				
1 (≤98.25)	33 (29.9)	28 (24.3)	1.00	1.00
2 (98.26–116.00)	28 (25.0)	31 (27.0)	1.10 (0.74–1.66)	0.87 (0.54–1.40)
3 (116.01–141.75)	27 (24.1)	29 (25.2)	0.91 (0.60–1.37)	0.84 (0.52–1.35)
4 (≥141.76)	29 (25.9)	27 (23.5)	1.02 (0.68–1.53)	1.02 (0.63–1.65)
Trend test			*P* =0.832	*P* = 0.991

Data in the table are adjusted OR and 95 % CI

^a^Adjusted for potential confounders including age, gender, education level

^b^Adjusted for age, gender, education level, BMI, hypertension, T2DM, and heart disease

## Discussion

In the present study, we explore the association between blood lipid/lipoprotein profiles and the risk of MCI. According to our results, after adjustment for some potential confounding factors, logistic regression models showed plasma TC was significantly higher in the MCI subjects compared to cognitively normal controls, while levels of HDL-C and TG were significantly lower. LDL-C levels did not differ significantly between two groups. Blood lipid levels are modifiable through diet, exercise, medications, and/or change in adverse lifestyle habits such as smoking. Therefore, all these results have an important policy implication. Strategies to intervene blood lipid/lipoprotein levels may be a viable population-wide intervention strategy to help maintain cognitive function with age.

Studies examining the role of plasma lipid levels in cognitive function reported inconsistent results [13, 20–22]. Controversial results have also been obtained in animal studies [23, 24]. Most observational studies were cross-sectional, the few longitudinal studies mostly examined manifest dementia but not MCI as the endpoint. While studies have found a relation between high cholesterol during mid-life and cognitive impairment or MCI in old age [21], the Honolulu Asia Aging Study recently reported in a study with 26 years of follow-up that cholesterol levels in men with dementia at the end of follow-up declined at least 15 years before the diagnosis and remained lower than cholesterol levels in men without dementia [25]. Associations relating late-life lipids with cognitive impairment or dementia were also inconsistent.

Plasma TC level differed significantly between MCI and control groups. TC were significantly higher in MCI group compared with the control. We initially observed that higher TC are associated with MCI adjusting for age, sex, and education, However, these associations were attenuated after adjusting for BMI, T2DM, heart disease and hypertension indicating that the initially observed inverse associations were caused by confounding. Notably, our findings are consistent with several previous studies. One cross-sectional population based study found that higher levels of TC were associated with a decreased risk of incident AD after adjustment for many confounding factors among the Finnish elderly aged 69 to 78 [26]. Prospective longitudinal studies also revealed that hypercholesterolaemia was associated with a protective effect for development of dementia and cognitive decline in the Australian elderly aged 75 years and over [27]. Elevated TC level was associated with a significantly higher risk of MCI, independent of potential confounding variables, suggesting that cholesterol fractions could be involved in both AD and MCI. The mechanism by which raised TC might lead to dementia is unclear. Recent evidence indicates that alterations in brain cholesterol homeostasis have been linked to the main pathological features of AD, in particular Aβ [28, 29]. Recent evidence suggests that amyloidogenic APP processing may preferentially occur in the cholesterol-rich regions of membranes known as lipid rafts, and that changes in cholesterol levels could exert their effects by altering the distribution of APP-cleaving enzymes within the membrane [30]. Functional cell biology studies further support a critical involvement of lipid raft cholesterol in the modulation of Aβ precursor protein processing by β-secretase and γ-secretase resulting in altered Aβ production. Reduction of cholesterol levels have been shown to inhibit β-secretase activity [31], but increase the activity of α-secretase [32], the main proteolytic enzymes involved in APP metabolism.

Moreover, the HDL-C level was significantly lower in MCI subjects compared to controls. Which is consistent with previous research findings [33–36]. Lower HDL-C level is associated with more severe lesions of white matter changes, leading to MCI, even AD [37]. The mechanisms associating low levels of HDL-C with cognitive impairment are unknown, and different explanations might be proposed. HDL-C has been described as a negative risk factor for the development of cognitive impairment [38]. HDL-C can prevent aggregation and polymerization of β-amyloid, thus slowing or even preventing the development of AD [39, 40]. HDL-C is also reported to have antiinflammatory properties [41]. Markers of inflammation are found in and around amyloid plaques and are considered to be important in the neurodegenerative process. These findings are of great clinical importance because they suggest that increasing HDL-C rather than lowering TC might prevent the development of cognitive impairment and dementia. Existing cardiovascular research suggests that HDL-C can be raised relatively easily through a diet containing monounsaturated and polyunsaturated fats, exercise, eliminating smoking, and using medications such as niacin, statins and fibrates among others when necessary [42]. New preventive and therapeutic strategies should identify the effects of HDL-C on cognitive function in the elderly.

Currently, there are very few studies on the direct association between cognitive function and TG. Among them, some studies show that the level of TG was lower in patients with cognitive impairment compared with control group [43, 44], while several found no relationship between TG and cognition [45], and others found that high levels of TG were inversely related with performance on various cognitive measures [46, 47]. In our study, high levels of TG were significantly related to lower odds of MCI. This suggests that higher level of TG are beneficial for cognitive function. A pathological concentration of TG may outweigh any protective effects. These results suggest that TG supplementation to high normal levels is worthy of study in clinical trials to determine whether it may improve cognitive function in older adults.

In our study, some limitations need to be addressed. First, this was a case-control study, and cannot prove causality. These findings need to be confirmed in further longitudinal studies. Second, our subjects were Chinese elderly, so these results may not be generalised to other populations of different nationalities or ages. Third, although group differences were observed for different lipid measures, the numerical difference between groups is small and there is substantial overlap. Perhaps, population type, diagnostic procedure, confounding control, presence of bias, statistical power can influence the accuracy and precision. To better capture the combined effects of these blood lipids on cognitive function, longitudinal analysis with multiple consistent lipoproteins and cognitive measures are needed from mid adulthood into older ages. The last limitation of the study is the lack of data for brain imaging or biomarker data (such as apolipoprotein E 4 allele) of the population sample. Therefore, a follow-up study, brain imaging, and biomarker tests will be conducted in the future.

Despite limitations, the chief strength of the present study include: First, extensively tested and well designed measurements of neuropsychiatric symptoms provided reliable diagnoses of MCI and dementia; Second, This is one of few studies which investigated the blood lipid/lipoprotein profiles related to cognitive impairment in a developing country, and adjusted for various important confounders including vascular risk factors. Third, the availability of pre-study plasma lipids/lipoproteins levels and other covariates.

## Conclusions

Based on the findings of the present study, it can be concluded that plasma TC was significantly higher in the patients with MCI compared to cognitively normal controls, while HDL-C and TG levels were significantly lower. LDL-C level did not differ significantly between two groups. Because associations are not proof of a causal relation, large randomized controlled clinical trials regarding blood lipid/lipoprotein profiles and the onset and course of MCI, AD are under way.

## Abbreviations

AD: Alzheimer’s disease
ADL: Activities of Daily Living scale
BMI: Body mass index
CIs: Confidence intervals
CSF: Cerebrospinal fluid
HDL-C: High-density lipoprotein cholesterol
LDL-C: Low-density lipoprotein cholesterol
MCI: Mild cognitive impairment
MMSE: Mini-Mental Status Examination
OR: Odds ratio
SD: standard deviation
T2DM: Type 2 diabetes millitus
TC: Total cholesterol
TG: Triglyceride
